# Impact of Oxygen Saturation on Mortality in Obese and Non-obese Critically Ill Patients With Mechanical Ventilation: A Retrospective Observational Study

**DOI:** 10.3389/fmed.2022.839787

**Published:** 2022-04-15

**Authors:** Tong Li, Dawei Zhou, Dong Zhao, Qing Lin, Dijia Wang, Chao Wang, Rongli Zhang

**Affiliations:** Department of Critical Care Medicine, Beijing Tongren Hospital, Capital Medical University, Beijing, China

**Keywords:** obesity, intensive care unit, mechanical ventilation, oxygen saturation, mortality

## Abstract

**Background:**

The main aim of this study was to evaluate the effect of oxygen saturation on mortality in critically ill patients with mechanical ventilation according to obesity status.

**Methods:**

We conducted an observational study in mechanically ventilated patients admitted to the ICU retrospectively. Demographic, arterial blood gas, ventilator setting, interventions, and peripheral oxygen saturation (Spo_2_) during the first 24 h were recorded and analyzed between non-obese and obese patients. The main exposure included Spo_2_, time-weighted mean Spo_2_ (TWM-Spo_2_), and proportion of time spent in different Spo_2_ (PTS-Spo_2_) levels. The primary outcome was hospital mortality. We used multivariable logistic regression models to assess the relationship between Spo_2_ and mortality, as well as the interaction between PTS-Spo_2_ and obesity status.

**Results:**

A total of 25,100 patients were included, of which 10,564 (42%) were obese patients. After adjusting for confounders, compared with TWM-Spo_2_ of 94–98%, TWM-Spo_2_ of < =88% (OR 3.572; CI [2.343, 5.455]; *p* < 0.001) and of 89–93% (OR 1.514; CI [1.343, 1.706]; *p* < 0.001) were both associated with higher risk of mortality. PTS-Spo_2_ of 99–100% was associated with increased risk of mortality for obese patients (OR 1.028; 95% CI 1.010–1.046; *p* = 0.002; P_interaction =_ 0.001), while PTS-Spo_2_ of 89–93% was associated with increased risk of mortality (OR 1.089; 95% CI 1.051–1.128; *p* < 0.001; P_interaction =_ 0.001) for non-obese patients.

**Conclusions:**

For obese and non-obese critically ill patients with mechanical ventilation, the impact of oxygen saturation on hospital mortality is different.

## Introduction

Obesity has emerged as one of the leading health concerns over the past century (1). In the United States, for the years 2013 and 2014, the overall obesity prevalence was 37.7% (2). Moreover, for adult obesity and severe obesity, the prevalence will continue to increase (3). Recent studies estimate by 2030 nearly 1 in 2 adults will have obesity (4, 5). At the same time, obese patients are over-represented in primary care (6). The similar circumstance is also reflected in critically ill patients where a recent study reported a prevalence of around 20% for the year 2012 (7, 8). Moreover, obese patients represent a specific population that seemed to be associated with high morbidity and increased resource utilization, who require an adapted ICU management (7–10).

The provision of supplemental oxygen is a ubiquitous intervention for mechanically ventilated patients. However, excessive oxygen could also be injurious (11, 12). British Thoracic Society (BTS) recommends, for morbidly obese patients who are at the risk of hypoventilation, even without evidence of coexistent obstructive sleep apnea, targeted oxygen saturation of 88–92% should be titrated (grade D) (13). Two small trials conducted in morbidly obese patients showed exposure to hyperoxia caused elevated partial pressure of carbon dioxide (Paco_2_) and hypoventilation (14, 15). However, for patients with milder obesity, the literature is really sparse, especially concerning the impact in the critical setting. Moreover, the BTS or Thoracic Society of Australia and New Zealand guidelines did not cover the area of ICU (13, 16). Given the epidemiologic and specific pathophysiologic changes observed in obese patients, one could hypothesize that the obesity status could be an important confounder in the relationship between supplemental oxygen and outcomes. To the best of our knowledge, no study has specifically evaluated the relationship between supplemental oxygen and hospital mortality outcome in obese critically ill patients with mechanical ventilation.

The main aim of this study was to analyze the influence of the obesity status on the relationship between oxygen saturation and the hospital mortality in mechanically ventilated patients. The hypothesis was the impact of the supplemental oxygen on mortality in non-obese and obese critically ill patients with mechanical ventilation may be different.

## Methods

### Setting

This study used data stored in the high-resolution eICU (eicu-crd.mit.edu) database, which comprises 200,859 admissions for 139,367 unique patients between 2014 and 2015 at 208 hospitals located throughout the United States. The database contains parameters that were available in the routine ICU clinical information system, including admission diagnosis, APACHE IV score and components, laboratory measurements, vital signs, medications, and special treatments. The elaborate description of eICU can be found elsewhere (17).

The eICU database was certified as meeting safe harbor standards by Privacert (Cambridge, MA) (Health Insurance Portability and Accountability Act Certification no. 1031219-2). The institutional review board (IRB) approval was exempt due to the retrospective and re-identification design. The author (certification number: 28795067) was approved to access the database for research aims after completing the National Institutes of Health web-based training course, which was “Protecting Human Research Participants.”

### Study Population

All recorded patients in the eICU database were eligible for inclusion. The first ICU stay was selected for those who were admitted to ICU for more than once. We selected adult patients with invasively mechanical ventilation during the first 24 h after admission to ICU. Patients were excluded for the following reasons: (1) ICU length of stay <24 h, (2) Incomplete hospital mortality recording, (3) Missing Spo_2_ data, (4) Percentage of recorded Spo_2_ < =50%, values of Spo_2_ <70% were excluded based on such values were likely to be not accurate, (5) Missing height or weight data, (6) Missing APACHE IV score, (7) Ventilation with ambient air, i.e., fraction of inspired oxygen (Fio_2_) = 21%.

### Clinical Variables

The following information data during the first 24 h of admission were extracted: age, gender, height, weight, ethnicity, comorbidities, admission diagnosis, ICU types, Acute Physiology and Chronic Health Evaluation (APACHE) IV score and components, and sequential organ failure assessment (SOFA) score. The use of dialysis, vasopressors, mechanical ventilation setting during the first 24 h were also collected. However, we could not extract the pattern of mechanical ventilation. Patients with a body mass index (BMI) ≥30 kg/(m^2^) were defined as obese according to the international standards (18), where BMI was calculated as body weight /(height^2^).

We included all arterial blood gas (ABG) samples that were obtained during the first 24 h admitted to the ICU. The time-weighted mean partial pressure of arterial oxygen (Pao_2_) (TWM- Pao_2_), Paco_2_ (TWM-Paco_2_), pH (TWM-pH), and Fio_2_ (TWM- Fio_2_) were calculated for every patient. The time-weighted mean (TWM) data was calculated as an area under the curve (AUC) by integrating all time-sequence data divided by the whole time (24 h). The duration of the first measured value represented the time from admission (0 h) to the first measurement time. The duration for the last measured value was the measurement time to 24 h in the ICU. TWM- Pao_2_, TWM-Paco_2_, and TWM-pH were considered as categorical variables, and patients with missing ABG data of Pao_2_, Paco_2_, and pH were considered as a unique category, respectively.

The Spo_2_, which was obtained during the first 24 h after ICU admission, were generally recorded from bedside vital signs monitors as 5-min median values. According to the BTS guideline for oxygen use in adults in healthcare and emergency settings (13), four categories were generated, which were < =88%, 89–93%, 94–98%, and 99–100%. TWM-Spo_2_ was calculated for every patient, and patients were classified into different Spo_2_ categories. We separately calculated the proportion of time spent in Spo_2_ (PTS-Spo_2_) of four categories during the first 24 h, defined as the time in each Spo_2_ category divided by total time. For each patient, PTS-Spo_2_ in each of the four predefined categories ranged from 0 to 100%, and the total proportion was 100%. A similar method was used in other studies (19, 20).

We selected the hospital mortality, defined as dead at hospital discharge as the primary outcome.

### Statistical Analysis

Categorical variables were reported as numbers and percentages and were analyzed with Chi-square test or Fisher's exact test as appropriate. Continuous variables are shown as median and interquartile range (IQR) or mean and standard deviation (SD), which were compared using Wilcoxon rank-sum test or Student's *t*-test, respectively. As for outliers in the database, which were defined as the recording lies outside the 3 × IQR range, were re-checked and replaced by the 5th or 95th percentile. As for missing values, the number was clearly stated for each variable. Patients with missing data of Pao_2_, Paco_2_, and pH were considered as a categorical variable, and multiple imputation was performed for missing data of TWM-Fio_2_.

First, a descriptive analysis was performed in non-obese and obese patients, including baseline characteristics and clinical parameters. Then, a univariate analysis was done according to survival at hospital discharge in non-obese and obese patients. We used the multivariable logistic regression models to assess the relationship between TWM-Spo_2_ categories and hospital mortality after adjusting for covariates. The association between each PTS-Spo_2_ of different categories and hospital mortality was investigated with multivariable logistic regression. The variables that were considered clinically relevant or that showed a univariate relationship with the outcome (*p* < 0.10) were selected into the multivariable model. A stepwise backward elimination method with a significance level of 0.05 was used to build the final model. The variance inflation factor was used to examine the multicollinearity, and the Hosmer-Lemeshow goodness-of-fit test to assess the calibration of the models.

PTS-Spo_2_ of different categories was compared between non-obese and obese patients. The interaction between PTS-Spo_2_ (as a continuous variable) and obesity status (obese and non-obese, as a categorical variable) was explored by the multivariable logistic regression model.

As for the data extraction, PostgreSQL (version 10, www.postgresql.org) was used. A two-sided *p*-value of <0.05 was considered statistically significant. We used the R software (version 3.5.1, www.r-project.org) to conduct all the statistical analyses.

## Results

**Figure 1:**
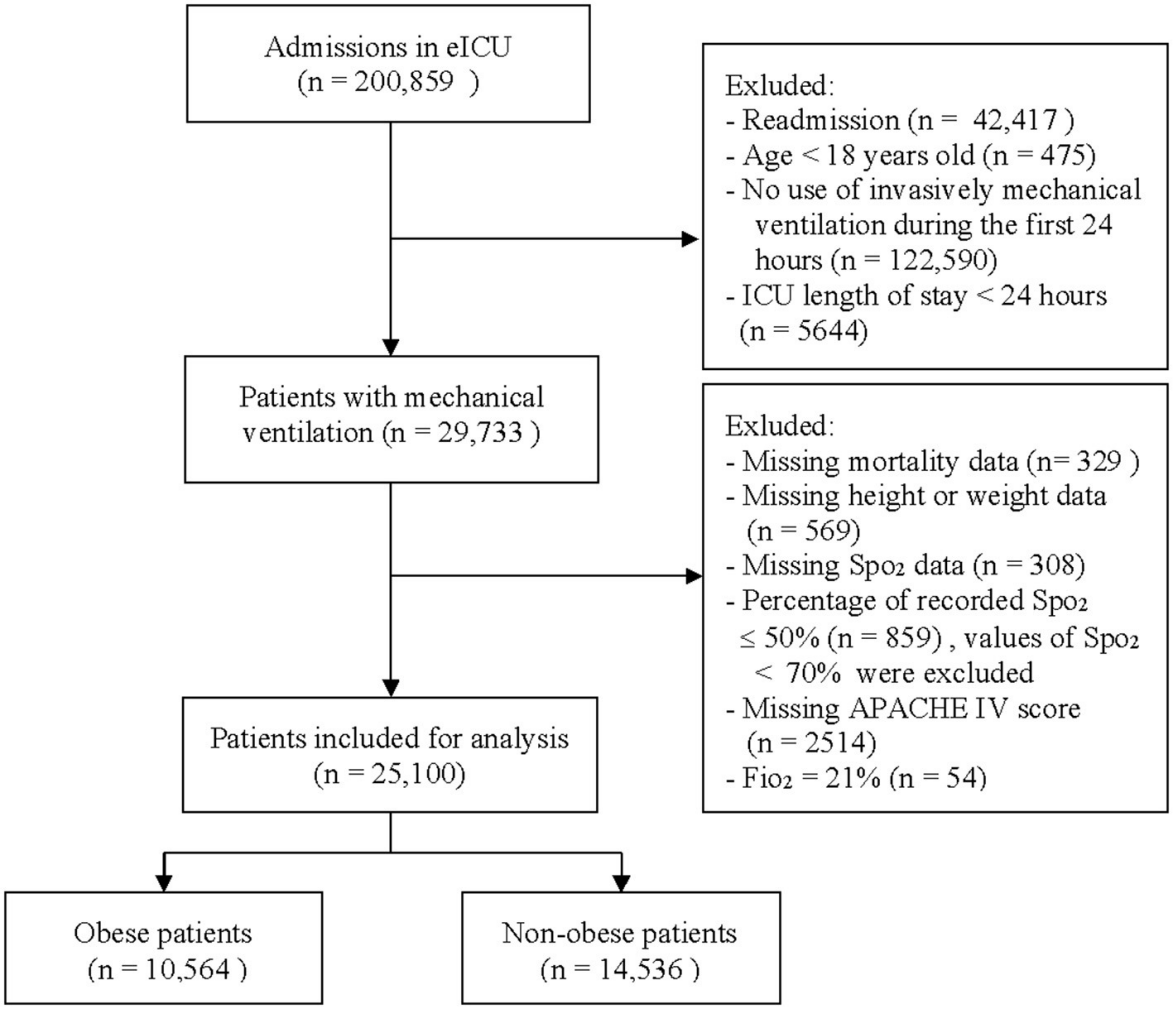
Flow chart of subject selection. eICU, telehealth intensive care unit; Spo_2_, peripheral oxygen saturation; Fio_2_, fraction of inspiration oxygen.

Figure 1 showed the flow chart of the study. After exclusion, a total of 25,100 patients met our inclusion criteria, of which 10,564 (42%) were obese patients and 14,534 (58%) were non-obese patients. The median BMI in the total population was 28.3 (IQR 23.9–34.4) kg/m^2^, of which the non-obese was 25 (IQR 22–27) kg/m^2^ and the obese was 36 (IQR 33–42) kg/m^2^. The median age was 65 (IQR 54–75) years, 13,632 (54%) was male. 4,242 (17%) patients were non-survivors at hospital discharge. Demographics and baseline characteristics between the obese and non-obese were presented in Table 1.

**Table 1:** Baseline characteristics of study patients.

**Variables**	**Total (*n =* 25,100)**		**Non-obese (*n =* 14,536)**		***P*-value**	**Obese (*n =* 10,564)**		***P*-value**
	**Non-obese (*n =* 14,536)**	**Obese (*n =* 10,564)**	**Survivors (*n =* 11,865)**	**Non-survivors (*n =* 2,671)**		**Survivors (*n =* 8,993)**	**Non-survivors (*n =* 1,571)**	
Age, years	67 (55, 78)	63 (53, 72)	66 (54, 77)	72 (60, 81)	<0.001	63 (53, 72)	67 (57, 75)	<0.001
Gender: male	8147 (56)	5485 (52)	6636 (56)	1511 (57)	0.561	4678 (52)	807 (51)	0.654
BMI	25 (22, 27)	36 (33, 42)	25 (22, 27)	24 (21, 27)	<0.001	36 (33, 42)	35 (32, 41)	0.014
Ethnicity					0.665			0.532
Caucasian	11154 (77)	8233 (78)	9083 (77)	2071 (78)		7005 (78)	1228 (78)	
African American	1602 (11)	1322 (13)	1322 (11)	280 (10)		1142 (13)	180 (11)	
Hispanic	549 (4)	325 (3)	448 (4)	101 (4)		276 (3)	49 (3)	
Asian	295 (2)	59 (1)	245 (2)	50 (2)		47 (1)	12 (1)	
Native American	85 (1)	101 (1)	74 (1)	11 (0)		84 (1)	17 (1)	
Other/Unknown	851 (6)	524 (5)	693 (6)	158 (6)		439 (5)	85 (5)	
**Comorbidities**
Hypertension	7095 (49)	6166 (58)	5722 (48)	1373 (51)	0.003	5267 (59)	899 (57)	0.333
Diabetes mellitus	2525 (17)	3471 (33)	2046 (17)	479 (18)	0.411	3000 (33)	471 (30)	0.009
COPD	3188 (22)	2560 (24)	2608 (22)	580 (22)	0.784	2205 (25)	355 (23)	0.108
Heart failure	2380 (16)	2525 (24)	1921 (16)	459 (17)	0.22	2098 (23)	427 (27)	0.001
Cirrhosis	252 (2)	180 (2)	192 (2)	60 (2)	0.03	136 (2)	44 (3)	<0.001
Chronic renal insufficiency	1879 (13)	1615 (15)	1480 (12)	399 (15)	0.001	1320 (15)	295 (19)	<0.001
ICU types					<0.001			<0.001
Med-SurgICU	7735 (53)	5691 (54)	6328 (53)	1407 (53)		4902 (55)	789 (50)	
Cardiac ICU	924 (6)	683 (6)	687 (6)	237 (9)		504 (6)	179 (11)	
CCU-CTICU	1168 (8)	942 (9)	982 (8)	186 (7)		818 (9)	124 (8)	
CSICU	516 (4)	366 (3)	441 (4)	75 (3)		321 (4)	45 (3)	
CTICU	662 (5)	445 (4)	608 (5)	54 (2)		409 (5)	36 (2)	
MICU	1446 (10)	1102 (10)	1144 (10)	302 (11)		912 (10)	190 (12)	
Neuro ICU	995 (7)	615 (6)	770 (6)	225 (8)		500 (6)	115 (7)	
SICU	1090 (7)	720 (7)	905 (8)	185 (7)		627 (7)	93 (6)	
Diagnosis					<0.001			<0.001
Respiratory	3231 (22)	2550 (24)	2711 (23)	520 (19)		2278 (25)	272 (17)	
Sepsis	2135 (15)	1448 (14)	1625 (14)	510 (19)		1170 (13)	278 (18)	
Cardiac surgery	1619 (11)	1257 (12)	1577 (13)	42 (2)		1217 (14)	40 (3)	
Neurological	1920 (13)	1129 (11)	1568 (13)	352 (13)		950 (11)	179 (11)	
Cardiovascular	1064 (7)	1041 (10)	895 (8)	169 (6)		916 (10)	125 (8)	
Cardiac arrest	1181 (8)	898 (9)	637 (5)	544 (20)		474 (5)	424 (27)	
Trauma	774 (5)	384 (4)	645 (5)	129 (5)		323 (4)	61 (4)	
Gastrointestinal	675 (5)	336 (3)	548 (5)	127 (5)		293 (3)	43 (3)	
Others	1937 (13)	1521 (14)	1659 (14)	278 (10)		1372 (15)	149 (9)	

*Data are median (interquartile range) or No/Total (%)*.

*BMI, body mass index; COPD, chronic obstructive pulmonary disease; ICU, intensive care unit; CCU, Coronary Care Unit; CTICU, Cardiothoracic ICU; CSICU, Cardiac Surgery ICU; MICU, Medical ICU; SICU, Surgical ICU*.

In non-obese patients, the median age was 67 (IQR 55–78) and the hospital mortality was 18%. BMI of non-obese patients was higher in survivors than in non-survivors (25 vs. 24, *p* < 0.001). Non-obese patients with comorbidities of hypertension or chronic renal insufficiency had higher hospital mortality (Table 1). Non-obese survivors had a higher percentage of TWM-Spo_2_ of 99–100% (Table 2).

**Table 2:** Clinical data during the first 24 h and interventions.

**Variables**	**Total (*n =* 25,100)**		**Non-obese (*n =* 14,536)**		***P*-value**	**Obese (*n =* 10,564)**		***P*-value**
	**Non-obese (*n =* 14,536)**	**Obese (*n =* 10,564)**	**Survivors (*n =* 11,865)**	**Non-survivors (*n =* 2,671)**		**Survivors (*n =* 8,993)**	**Non-survivors (*n =* 1,571)**	
TWM-Spo_2_					<0.001			<0.001
< =88%	71 (0)	58 (1)	22 (0)	49 (2)		37 (0)	21 (1)	
89–93%	1016 (7)	1341 (13)	740 (6)	276 (10)		1118 (12)	223 (14)	
94-98%	10249 (71)	7994 (76)	8476 (71)	1773 (66)		6889 (77)	1105 (70)	
99–100%	3200 (22)	1171 (11)	2627 (22)	573 (21)		949 (11)	222 (14)	
TWM-Pao_2_, (mmHg)					<0.001			<0.001
<60	368 (3)	327 (3)	274 (2)	94 (4)		270 (3)	57 (4)	
60–120	5269 (36)	5139 (49)	4285 (36)	984 (37)		4381 (49)	758 (48)	
120–300	5199 (36)	2718 (26)	4203 (35)	996 (37)		2239 (25)	479 (30)	
>300	403 (3)	155 (1)	320 (3)	83 (3)		120 (1)	35 (2)	
Missing, *N* (%)	3297 (23)	2225 (21)	2783 (23)	514 (19)		1983 (22)	242 (15)	
TWM-Paco_2_, (mmHg)					<0.001			<0.001
<35	2951 (20)	1387 (13)	2240 (19)	711 (27)		1040 (12)	347 (22)	
35–45	5549 (38)	3799 (36)	4622 (39)	927 (35)		3245 (36)	554 (35)	
>45	2618 (18)	3079 (29)	2129 (18)	489 (18)		2667 (30)	412 (26)	
Missing, *N* (%)	3418 (24)	2299 (22)	2874 (24)	544 (20)		2041 (23)	258 (16)	
TWM-pH					<0.001			<0.001
<7.35	3579 (25)	3132 (30)	2641 (22)	938 (35)		2547 (28)	585 (37)	
7.35–7.45	5783 (40)	4069 (39)	4920 (41)	863 (32)		3513 (39)	556 (35)	
>7.45	1633 (11)	941 (9)	1318 (11)	315 (12)		779 (9)	162 (10)	
Missing, *N* (%)	3541 (24)	2422 (23)	2986 (25)	555 (21)		2154 (24)	268 (17)	
**Mechanical ventilation setting**
Fio_2_, %	50 (40, 60)	55 (40, 70)	50 (40, 60)	60 (50, 80)	<0.001	50 (40, 70)	60 (50, 80)	<0.001
RR	15 (12, 18)	16 (14, 20)	14 (12, 18)	16 (14, 20)	<0.001	16 (14, 18)	18 (14, 23)	<0.001
Tidal volume, ml/kg	6.7 (6.9, 7.6)	7 (6.3, 8.2)	6.7 (5.9, 7.7)	6.6 (5.8, 7.5)	<0.001	7.1 (6.3, 8.3)	7 (6.3, 8)	0.005
PEEP, cmH_2_O	5 (5, 5)	5 (5, 7)	5 (5, 5)	5 (5, 8)	<0.001	5 (5, 7)	5 (5, 8)	<0.001
Plateau pressure, cmH_2_O	19 (16, 24)	22 (18, 27)	19 (15, 23)	21 (17, 26)	<0.001	22 (18, 27)	24 (20, 29)	<0.001
APACHE-IV	69 (52, 90)	65 (49, 87)	65 (49, 84)	92 (72, 113)	<0.001	62 (47, 81)	94 (71, 118)	<0.001
SOFA	6 (4, 9)	6 (4, 8)	6 (4, 8)	8 (6, 11)	<0.001	6 (4, 8)	9 (6, 11)	<0.001
Vasopressors	3361 (23)	2323 (22)	2377 (20)	984 (37)	<0.001	1725 (19)	598 (38)	<0.001
Dialysis	601 (4)	351 (3)	499 (4)	102 (4)	0.393	283 (3)	68 (4)	0.02
Total ventilation days	3 (2, 5)	3 (2, 6)	2 (2, 4)	4 (2, 7)	<0.001	3 (2, 5)	4 (3, 8)	<0.001
Duration of ICU stay	3 (2, 6)	3 (2, 7)	3 (2, 6)	4 (2, 7)	<0.001	3 (2, 6)	4 (2, 8)	<0.001
Duration of hospital stay	8 (5, 13)	8 (5, 14)	8 (5, 14)	6 (3, 11)	<0.001	8 (5, 14)	6 (3, 11)	<0.001

*Data are median (interquartile range) or No/Total (%)*.

*TWM, time-weighted mean; Spo_2_, peripheral oxygen saturation; Pao_2_, partial pressure of arterial oxygen; Paco_2_, partial pressure of arterial carbon dioxide; Fio_2_, fraction of inspired oxygen; RR, respiratory rate; PEEP, positive end-expiratory pressure; APACHE, Acute Physiology, and Chronic Health Evaluation; SOFA, sequential organ failure assessment*.

In obese patients, the median age was 63 (IQR 53–72) and hospital mortality was 15%, which was lower than non-obese patients (*p* < 0.001). Obese survivors had higher BMI than non-survivors (36 vs. 35, *p* = 0.014). Obese patients had a higher percentage of hypertension, diabetes mellitus, chronic heart failure, chronic obstructive pulmonary disease, and chronic renal insufficiency than the non-obese. Interestingly, obese survivors had a higher percentage of diabetes mellitus (33% vs. 30%, *p* = 0.009). Obese survivors had lower percentage of TWM-Spo_2_ of 99–100% (11% vs. 14%) and higher percentage of TWM-Paco_2_ > 45 mmHg (30 vs. 26%) (Table 2). Obese patients had lower PTS-Spo_2_ of 99–100% than non-obese patients (39.8% vs. 54.8%, *p* < 0.001) (Table 3).

**Table 3:** Adjusted odds ratio for hospital mortality in patients with different PTS-Spo_2_ categories.

**Proportion of time (%) spent in Spo_**2**_ categories**	**Non-obese (*n =* 14,536)**	**Obese (*n =* 10,564)**	**Adjusted ORs for hospital mortality (95% CI)**	***P*-Value**	***P* for interaction**
< =88%, median (IQR)	0 (0, 0.8)	0 (0, 1.1)	1.570 [1.435, 1.723]	<0.001	0.01
89–93%, median (IQR)	1.3 (0, 7.8)	3.4 (0.4, 14.3)	1.100 [1.062, 1.138]	<0.001	0.001
94–98%, mean (SD)	37 (28.3)	47.6 (27.8)	0.977 [0.961, 0.994]	0.008	0.007
99–100%, mean (SD)	54.8 (34)	39.8 (33.3)	1.000 [0.986, 1.014]	0.985	0.001

*PTS, proportion of time spent; Spo_2_, peripheral oxygen saturation; SD, standard deviation; OR, odd ratio; CI, confidence interval*.

*All models included age, BMI categories (obese and non-obese), admission diagnosis, diabetes mellitus, Sequential Organ Failure Assessment score (not including points for the respiratory part), TWM-Fio_2_, TWM-Paco_2_ categories, and TWM-pH categories as covariates. Four categories (i.e., four multivariable logistic models including four categories and other covariates) of proportion of time spent in Spo_2_ were selected into the models separately, and the interaction between proportion of time spent in Spo_2_ and BMI categories were explored. BMI, body mass index; TWM, time-weighted mean; Fio2, fraction of inspiration oxygen; Paco2, partial pressure of arterial carbon dioxide*.

*An odd ratio is calculated per 10% increase in time in each given band*.

**Figure 2:**
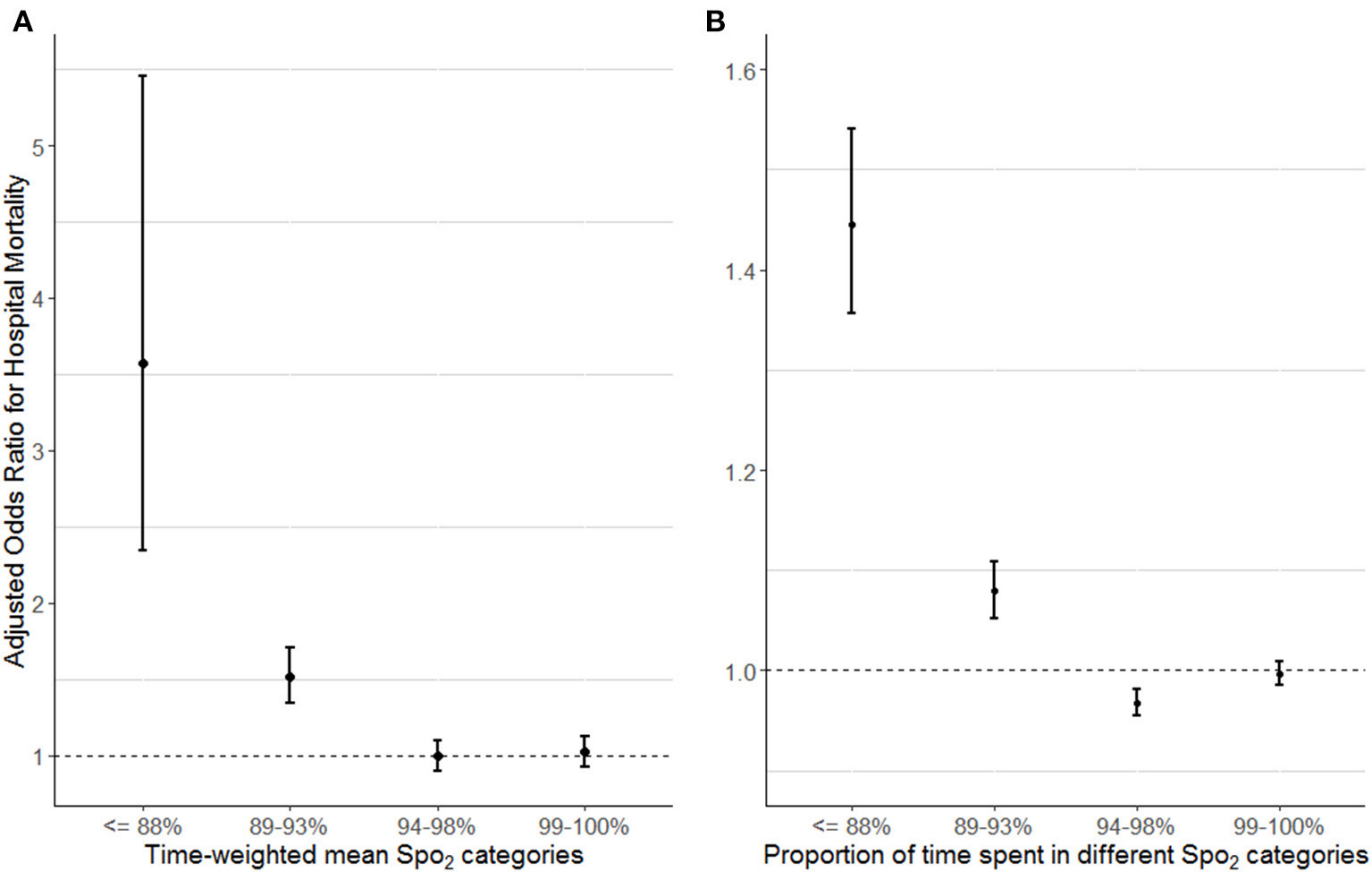
Left **(A)** Adjusted odds ratio for hospital mortality according to different TWM-Spo_2_ categories. The adjusted odds ratio for each Spo_2_ level and 95% confidence intervals (error bars) were calculated (TWM-Spo_2_ of 94–98% as reference). Right **(B)** Adjusted odds ratio for hospital mortality according to PTS-Spo_2_ categories separately. An odds ratio is calculated per 10% increase in time in each given category. All models (i.e., five models, one for “**A**” and four for “**B**”) were separately adjusted for age, BMI categories, admission diagnosis, diabetes mellitus, Sequential Organ Failure Assessment score (not including points for the respiratory part), TWM-Fio_2_, TWM-Paco_2_, and TWM-pH categories. TWM, time-weighted mean; Spo_2_, peripheral oxygen saturation; PTS, proportion of time spent; BMI, body mass index; Fio_2_, fraction of inspiration oxygen; Paco_2_, partial pressure of arterial carbon dioxide. Full multivariable models see Supplementary Tables 1–5.

The relationship between TWM-Spo_2_ categories and hospital mortality, which was assessed by the multivariable logistic models after adjusting confounders, was comparing with 94–98% category, < =88% category (OR 3.572; CI [2.343, 5.455]; *p* < 0.001) and 89–93% category (OR 1.514; CI [1.343, 1.706]; *p* < 0.001) both had higher odds ratio for hospital mortality, while 99–100% category was not associated with hospital mortality (Figure 2A and Supplementary Table 1). After multivariable analysis, PTS-Spo_2_ of < =88% (OR 1.445; CI [1.356, 1.541]; *p* < 0.001; per 10% increase) and PTS-Spo_2_ of 89–93% (OR 1.080; CI [1.052, 1.108]; *p* < 0.001; per 10% increase) had higher odds ratio for hospital mortality, PTS-Spo_2_ of 94–98% (OR 0.968; CI [0.955, 0.981]; *p* < 0.001; per 10% increase) had lower odd ratio for hospital mortality, and PTS-Spo_2_ of 99–100% (OR 0.997; CI [0.986, 1.009]; *p* = 0.636; per 10% increase) was not associated with high risk of hospital mortality (Figure 2B and Supplementary Tables 2–5).

**Figure 3:**
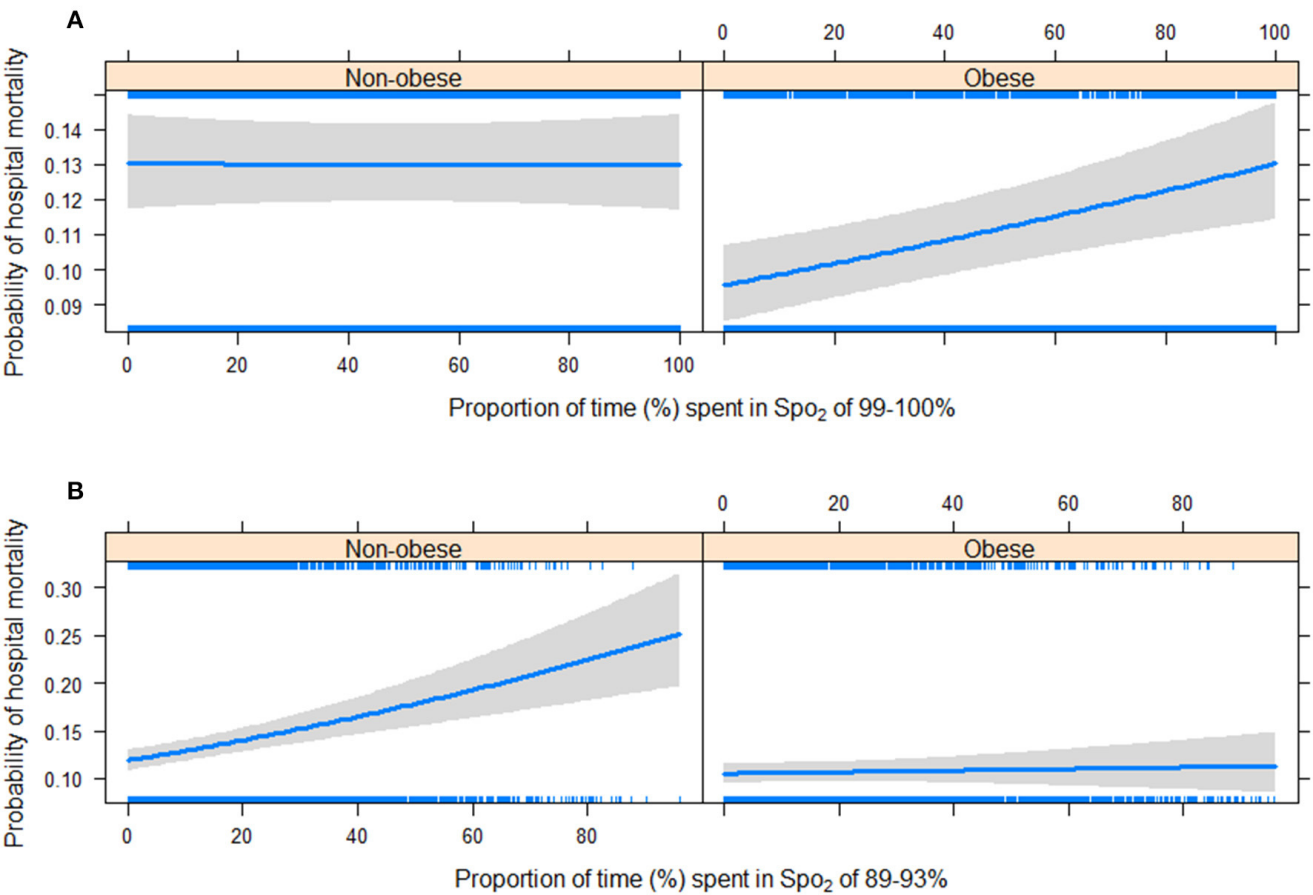
Interaction between PTS-Spo_2_ and obesity status (obese and non-obese). Upper **(A)** While the PTS-Spo_2_ of 99–100% was not significantly associated with hospital mortality outcome (OR 1.004; 95% CI 0.990–1.018; *p* = 0.597; per 10% increase) in non-obese patients, it was associated with increased risk of mortality in obese patients (OR 1.028; 95% CI 1.010–1.046; *p* = 0.002; per 10% increase). Lower **(B)** While the PTS-Spo_2_ of 89–93% was significantly associated with increased risk of hospital mortality outcome (OR 1.089; 95% CI 1.051–1.128; *p* < 0.001; per 10% increase) in non-obese patients, it was not associated with hospital mortality in obese patients (OR 1.022; 95% CI 0.985–1.060; *p* = 0.235; per 10% increase). PTS-Spo_2_, proportion of time spent in peripheral oxygen saturation, OR odds ratio, CI confidential interval.

The multivariable models including the interaction between PTS-Spo_2_ and obesity status (non-obese and obese) showed that there was a significant interaction between PTS-Spo_2_ and obesity status (Table 3). While the PTS-Spo_2_ of 99-100% was not significantly associated with hospital mortality outcome (OR 1.004; 95% CI 0.990-1.018; p = 0.597; per 10% increase) in non-obese patients, it was associated with increased risk of mortality in obese patients (OR 1.028; 95% CI 1.010-1.046; p = 0.002; per 10% increase) (Figure 3A). While the PTS-Spo_2_ of 89-93% was significantly associated with increased risk of hospital mortality outcome (OR 1.089; 95% CI 1.051-1.128; p <0.001; per 10% increase) in non-obese patients, it was not associated with hospital mortality in obese patients (OR 1.022; 95% CI 0.985-1.060; p = 0.235; per 10% increase) (Figure 3B). Supplementary Figure 3 showed the interaction between PTS-Spo_2_ of < = 88% and 94-98% and obesity status (obese and non-obese).

## Discussion

The results of our study provide evidence to support our hypothesis. The impact of supplemental oxygen on hospital mortality was different in non-obese and obese critically ill patients with mechanical ventilation. Spo_2_ of 99–100% was associated with a higher hospital mortality for obese patients but was not statistically significant for non-obese patients. Spo_2_ of 94–98% was associated with decreased risk of hospital mortality outcome for both obese and non-obese patients. Spo_2_ of 89–93% was associated with higher mortality for non-obese patients, but not statistically significant for obese patients. Spo_2_ of < =88% was associated with an increased risk of hospital mortality outcome in both non-obese patients and obese patients. This is the first study to assess the relationship between supplemental oxygen and mortality in the specific population of obese critically ill patients with mechanical ventilation. The results of the present study showed that supplemental oxygen therapy should consider the obesity status into account.

The Oxygen-ICU randomized clinical trial showed among critically ill patients with an ICU length of stay of 72 h or longer, conservative therapy (Spo_2_ 94–98%) vs. conventional therapy (Spo_2_ 97–100%) resulted in lower ICU mortality (11). However, ICU-ROX trial found in adults undergoing mechanical ventilation in the ICU, conservative oxygen therapy (Spo_2_ 91–96%), as compared with usual oxygen therapy (Spo_2_ ≥91%), did not significantly affect the number of ventilator-free days or mortality (21). In the present study, compared with the TWM-Spo_2_ 94–98% category, TWM-Spo_2_ of 99–100% was not associated with hospital mortality. However, when considering the dose of Spo_2_, PTS-Spo_2_ of 94–98% was associated with lower hospital mortality in overall patients. The results of our study were consistent with the two RCTs.

There were some explanations of the differences observed between the non-obese and obese groups. First, obese patients can cause hypoxemia by decreasing lung volumes and may be at risk for hyperoxia-induced hypercapnia (22, 23). Two small trials conducted in morbidly obese patients with obesity hypoventilation syndrome (OHS) showed an elevated baseline Paco_2_. After a 20-min exposure to hyperoxia, the patients had a mean increase in Paco_2_ of about 4.4 mmHg (14, 15). However, whether hyperoxia-induced hypercapnia occurs in patients with milder obesity and without hypoventilation is unclear. Denault et al. found in obese patients with mean BMI 34 kg/m^2^ after cardiac surgery, there was no clinically important increase in Paco_2_ associated with higher Spo_2_ values (24). Second, obese patients had higher rate of comorbidities, like COPD, chronic heart failure, diabetes mellitus, of which hyperoxia could exacerbate the outcome (25, 26). In the present study, percentage of COPD or heart failure was higher in obese than non-obese patients. Interestingly, in obese patients, survivors had a higher percentage of diabetes mellitus than non-survivors, which was not the case in the non-obese patients. Vught LA et al. also found diabetes was not associated with adjusted 90-day mortality risk in critically ill patients admitted with sepsis (27). Third, hyperoxia may result in nitric oxide depletion and induction of oxidative stress on the adipose tissue, which may exacerbate the critical illness (28).

The results of the current study suggest that compared with non-obese patients, excessive oxygen may be injurious for obese patients. However, targeted oxygenation was still unknown. BTS guideline recommends for morbidly obese patients (BMI > 40 kg/m_2_), a target saturation of 88–92% should be titrated to maintain (grade D) (13). However, the literature was really sparse. In the present study, for obese patients with mechanical ventilation, Spo_2_ of < =88% may be associated with poor outcome, 89–93% was not associated with mortality, 94–98% was associated with lower mortality, and 99–100% was associated with higher mortality. The results suggested Spo_2_ 99–100% was not appropriate for obese patients. On the other hand, in obese patients, non-survivors had a higher percentage of TWM-Pao_2_ of 120–300 mmHg and >300 mmHg, which also suggested hyperoxia may be detrimental for obese patients.

For overall and non-obese patients, PTS-Spo_2_ of 89–93% was associated with increased hospital mortality in the present study. The contribution of modest hypoxemia to mortality is unknown. The recommendation of oxygen supply by guidelines mainly based on the normal ranges of Spo_2_ (13, 16). A North American study found Spo_2_ at sea level for adults with two standard deviation range is 94–98% (29). However, the standard reference ranges obtained from healthy volunteers may differ from the analogous range generated from data of ICU patients (30). Another larger observational study of over 37,000 patients admitted to four acute medical admissions units found that median Spo_2_ was 98% (IQR 97–99%) for young adults and 96% (IQR 95–98%) for old adults (31). They also found for patients with initial Spo_2_ values of 89, 90, 91, 92, and 93% had higher mortality and the 95% confidential intervals did not overlap with those initial Spo_2_ values of ≥95% (31). However, clearly, there were limitations to the raw results without adjusting for confounders.

The present study has several other strengths. First, a total of 25,100 patients was extracted from ICUs in 166 hospitals, which allowed for the adjustment of multiple confounding factors, making the findings more generalizable. Second, the total observations of Spo_2_ were 6,925,863, the average observations of Spo_2_ were 276 for each patient, accounting for the time of 95.8% in the first 24 h, which made the results robust. Third, we calculated both the TWM-Spo_2_ and PTS-Spo_2_, which considered the dose of Spo_2_.

However, several obvious limitations should be mentioned. First, the retrospective design in nature was subjected to the inherent limitations. This study design may only show statistical associations and but not causality between PTS-Spo_2_ and hospital mortality. Second, although the multivariable logistic regression to was used to adjust for potential confounders, the potential confounding factors (e.g., altitude, smoking status et al.) which were not included in the analysis, could lead to biased results. Third, some data could be invalidated due to the acquisition directly from the bedside vital sign monitors. Some of the data had not been checked and verified by a bedside care provider, i.e., the measurements may be noisy and may not reflect the true status. However, the Spo_2_ values recorded by the nurse and observed by the monitor at the same time were highly correlated (Supplementary Figure 4). Fourth, we only extracted the data for the first 24 h, which could not represent the entire course of stay in ICU. Fifth, we arbitrarily classified the Spo_2_ into four categories, < =88%, 89–93, 94–98%, and 99–100%, the rationality may need further research. Sixth, although Spo_2_ is widely used for clinical practice for continuously assessing the oxygenation in critically ill patients, blood gasses analyses are still the golden standard, especially in assessing the severity of hypoxemia and other gas exchange anomalies. Spo_2_ may not display the real oxygenation status. Finally, we excluded patients without APACHE IV score, whose diagnoses were mainly burns and certain organ transplantation (17, 32), which may cause biases.

## Conclusions

The impact of supplemental oxygen on hospital mortality was different in non-obese and obese critically ill patients with mechanical ventilation. Spo_2_ of 99–100% was associated with higher mortality for obese patients, while Spo_2_ of 89–93% was associated with higher mortality for non-obese patients.

## Data Availability Statement

Publicly available datasets were analyzed in this study. This data can be found here: eicu-crd.mit.edu.

## Ethics Statement

The eICU was exempt from Institutional Review Board (IRB) approval due to the retrospective design, lack of direct patient intervention, and the security schema, for which the re-identification risk was certified as meeting safe harbor standards by Privacert (Cambridge, MA) (Health Insurance Portability and Accountability Act Certification no. 1031219-2). Due to the HIPAA compliant de-identification in this database, our institutional IRB requirement was waived.

## Author Contributions

DZ and TL conceived this study and reviewed and modified the final manuscript. DZ extracted the data. TL, DZ, DW, and CW designed and performed the statistical analyses. DZ, TL, QL, RZ, and DZ wrote the first draft of the manuscript. All authors read, critically reviewed, and approved the final manuscript.

## Funding

This study was supported by Youth Top-notch Talent Program of Beijing Tongren Hospital.

## Conflict of Interest

The authors declare that the research was conducted in the absence of any commercial or financial relationships that could be construed as a potential conflict of interest.

## Publisher's Note

All claims expressed in this article are solely those of the authors and do not necessarily represent those of their affiliated organizations, or those of the publisher, the editors and the reviewers. Any product that may be evaluated in this article, or claim that may be made by its manufacturer, is not guaranteed or endorsed by the publisher.
