# Supplementary material for: Impact of Oxygen Saturation on Mortality in Obese and Non-obese Critically Ill Patients With Mechanical Ventilation: A Retrospective Observational Study
Source: Front Med (Lausanne). 2022 Apr 15;9:839787. doi: 10.3389/fmed.2022.839787 (PMC9051400; doi:10.3389/fmed.2022.839787)
Supplement: Supplementary file 1 [file Data_Sheet_1.DOCX]

**Impact of the oxygen saturation on mortality in obese and non-obese critically ill patients with mechanical ventilation: A retrospective observational study**

**Supplementary Material**

Tong Li^1^, Dawei Zhou^1^, Dong Zhao^1^, Qing Lin^1^, Dijia Wang^1^, Chao Wang^1^,

Rongli Zhang^1^

1: Department of Critical Care Medicine, Beijing Tongren Hospital, Capital Medical University, Beijing, China

| Variables | Multivariable Logistic Regression Analysis | |
| --- | --- | --- |
|  | OR (95% CI) | P value |
| TWM-Spo_2_ |  |  |
| 94-98% | 1 [Reference] |  |
| <= 88% | 3.572 [2.343, 5.455] | < 0.001 |
| 89-93% | 1.514 [1.343, 1.706] | < 0.001 |
| 99-100% | 1.021 [0.926, 1.124] | 0.679 |
| Age | 1.027 [1.024, 1.029] | < 0.001 |
| BMI categories |  |  |
| Normal weight | 1 [Reference] |  |
| Underweight | 1.189 [1.001, 1.407] | 0.046 |
| Overweight | 0.861 [0.782, 0.948] | 0.002 |
| Obese grade 1 | 0.784 [0.701, 0.875] | < 0.001 |
| Obese grade 2 | 0.781 [0.681, 0.895] | < 0.001 |
| Obese grade 3 | 0.760 [0.666, 0.867] | < 0.001 |
| Diagnosis |  |  |
| Cardiac arrest | 1 [Reference] |  |
| Respiratory | 0.271 [0.239, 0.307] | < 0.001 |
| Sepsis | 0.339 [0.299, 0.385] | < 0.001 |
| Cardiac surgery | 0.052 [0.041, 0.066] | < 0.001 |
| Neurological | 0.431 [0.375, 0.494] | < 0.001 |
| Cardiovascular | 0.254 [0.216, 0.299] | < 0.001 |
| Trauma | 0.457 [0.376, 0.553] | < 0.001 |
| Gastrointestinal | 0.280 [0.229, 0.340] | < 0.001 |
| Others | 0.244 [0.212, 0.281] | < 0.001 |
| Diabetes mellitus (yes vs no) | 0.870 [0.796, 0.951] | 0.002 |
| TWM-Fio_2_ (every 10% increase) | 1.159 [1.136, 1.182] | < 0.001 |
| TWM-pH |  |  |
| Normal pH | 1 [Reference] |  |
| Acidosis | 1.319 [1.205, 1.444] | < 0.001 |
| Alkalosis | 1.174 [1.038, 1.327] | 0.011 |
| pH missing | 0.922 [0.834, 1.018] | 0.108 |
| TWM-Paco_2_, mmHg |  |  |
| 35-45 | 1 [Reference] |  |
| < 35 | 1.569 [1.421, 1.733] | < 0.001 |
| > 45 | 0.759 [0.683, 0.842] | < 0.001 |
| Paco_2_ missing | 0.955 [0.754, 1.211] | 0.7 |
| SOFA score (not including respiratory part) | 1.205 [1.189, 1.221] | < 0.001 |

Supplementary table 1 Full multivariable logistic model assessing the impact of TWM-Spo_2_ on hospital mortality

TWM time-weighted mean, Spo_2_ peripheral oxygen saturation, BMI, body mass index, Fio_2_ fraction of inspired oxygen, Paco_2_ partial pressure of arterial carbon dioxide, SOFA sequential organ failure assessment.

BMI categories: Underweight: BMI < 18.5 kg/m^2^, Normal weight: 18.5 kg/m^2^ ≤ BMI < 25 kg/m^2^, Overweight: 25 kg/m^2^ ≤ BMI < 30 kg/m^2^, Obesity grade 1: 30 kg/m^2^ ≤ BMI < 35 kg/m^2^, Obesity grade 2: 35 kg/m^2^ ≤ BMI < 40 kg/m^2^, Obesity grade 3: 40 kg/m^2^ ≤ BMI.

Supplementary table 2 Full multivariable logistic model assessing the impact of PTS-Spo_2_ of <= 88% on hospital mortality

| Variables | Multivariable Logistic Regression Analysis | |
| --- | --- | --- |
|  | OR (95% CI) | P value |
| PTS-Spo_2_ of <= 88% (per 10% increase) | 1.445 [1.356, 1.541] | <0.001 |
| Age | 1.026 [1.024, 1.029] | <0.001 |
| BMI categories |  |  |
| Normal weight | 1 [Reference] |  |
| Underweight | 1.180 [0.994, 1.397] | 0.057 |
| Overweight | 0.873 [0.793, 0.961] | 0.006 |
| Obese grade 1 | 0.797 [0.714, 0.890] | <0.001 |
| Obese grade 2 | 0.800 [0.698, 0.917] | 0.001 |
| Obese grade 3 | 0.777 [0.682, 0.885] | <0.001 |
| Diagnosis |  |  |
| Cardiac arrest | 1 [Reference] |  |
| Respiratory | 0.274 [0.242, 0.311] | <0.001 |
| Sepsis | 0.342 [0.301, 0.388] | <0.001 |
| Cardiac surgery | 0.054 [0.042, 0.068] | <0.001 |
| Neurological | 0.436 [0.380, 0.501] | <0.001 |
| Cardiovascular | 0.256 [0.218, 0.300] | <0.001 |
| Trauma | 0.462 [0.380, 0.559] | <0.001 |
| Gastrointestinal | 0.284 [0.233, 0.346] | <0.001 |
| Others | 0.247 [0.214, 0.284] | <0.001 |
| Diabetes mellitus (yes vs no) | 0.873 [0.798, 0.953] | 0.003 |
| TWM-Fio_2_ (every 10% increase) | 1.152 [1.129, 1.175] | <0.001 |
| TWM-pH |  |  |
| Normal pH | 1 [Reference] |  |
| Acidosis | 1.299 [1.187, 1.422] | <0.001 |
| Alkalosis | 1.171 [1.035, 1.323] | 0.012 |
| pH missing | 0.920 [0.833, 1.016] | 0.102 |
| TWM-Paco_2_, mmHg |  |  |
| 35-45 | 1 [Reference] |  |
| < 35 | 1.547 [1.401, 1.708] | < 0.001 |
| > 45 | 0.764 [0.689, 0.848] | < 0.001 |
| Paco_2_ missing | 0.945 [0.747, 1.199] | 0.639 |
| SOFA score (not including respiratory part) | 1.202 [1.186, 1.218] | <0.001 |

PTS proportion of time spent, Spo_2_ peripheral oxygen saturation, BMI, body mass index, TWM time-weighted mean, Fio_2_ fraction of inspired oxygen, Paco_2_ partial pressure of arterial carbon dioxide, SOFA sequential organ failure assessment.

BMI categories: Underweight: BMI < 18.5 kg/m^2^, Normal weight: 18.5 kg/m^2^ ≤ BMI < 25 kg/m^2^, Overweight: 25 kg/m^2^ ≤ BMI < 30 kg/m^2^, Obesity grade 1: 30 kg/m^2^ ≤ BMI < 35 kg/m^2^, Obesity grade 2: 35 kg/m^2^ ≤ BMI < 40 kg/m^2^, Obesity grade 3: 40 kg/m^2^ ≤ BMI.

Supplementary table 3 Full multivariable logistic model assessing the impact of PTS-Spo_2_ of 89-93% on hospital mortality

| Variables | Multivariable Logistic Regression Analysis | |
| --- | --- | --- |
|  | OR (95% CI) | P value |
| PTS-Spo_2_ of 89-93% (per 10% increase) | 1.080 [1.052, 1.108] | <0.001 |
| Age | 1.026 [1.024, 1.029] | <0.001 |
| BMI categories |  |  |
| Normal weight | 1 [Reference] |  |
| Underweight | 1.200 [1.011, 1.421] | 0.035 |
| Overweight | 0.864 [0.785, 0.951] | 0.003 |
| Obese grade 1 | 0.778 [0.697, 0.868] | <0.001 |
| Obese grade 2 | 0.778 [0.678, 0.891] | <0.001 |
| Obese grade 3 | 0.759 [0.665, 0.864] | <0.001 |
| Diagnosis |  |  |
| Cardiac arrest | 1 [Reference] |  |
| Respiratory | 0.269 [0.237, 0.305] | <0.001 |
| Sepsis | 0.340 [0.299, 0.385] | <0.001 |
| Cardiac surgery | 0.051 [0.040, 0.065] | <0.001 |
| Neurological | 0.431 [0.375, 0.494] | <0.001 |
| Cardiovascular | 0.252 [0.215, 0.296] | <0.001 |
| Trauma | 0.459 [0.378, 0.555] | <0.001 |
| Gastrointestinal | 0.280 [0.230, 0.341] | <0.001 |
| Others | 0.242 [0.210, 0.279] | <0.001 |
| Diabetes mellitus (yes vs no) | 0.867 [0.793, 0.947] | 0.002 |
| TWM-Fio_2_ (every 10% increase) | 1.162 [1.140, 1.186] | <0.001 |
| TWM-pH |  |  |
| Normal pH | 1 [Reference] |  |
| Acidosis | 1.328 [1.214, 1.453] | <0.001 |
| Alkalosis | 1.180 [1.043, 1.333] | 0.008 |
| pH missing | 0.925 [0.837, 1.022] | 0.125 |
| TWM-Paco_2_, mmHg |  |  |
| 35-45 | 1 [Reference] |  |
| < 35 | 1.589 [1.440, 1.754] | < 0.001 |
| > 45 | 0.759 [0.684, 0.842] | < 0.001 |
| Paco_2_ missing | 0.953 [0.753, 1.208] | 0.64 |
| SOFA score (not including respiratory part) | 1.207 [1.191, 1.223] | <0.001 |

PTS proportion of time spent, Spo_2_ peripheral oxygen saturation, BMI, body mass index, TWM time-weighted mean, Fio_2_ fraction of inspired oxygen, Paco_2_ partial pressure of arterial carbon dioxide, SOFA sequential organ failure assessment.

BMI categories: Underweight: BMI < 18.5 kg/m^2^, Normal weight: 18.5 kg/m^2^ ≤ BMI < 25 kg/m^2^, Overweight: 25 kg/m^2^ ≤ BMI < 30 kg/m^2^, Obesity grade 1: 30 kg/m^2^ ≤ BMI < 35 kg/m^2^, Obesity grade 2: 35 kg/m^2^ ≤ BMI < 40 kg/m^2^, Obesity grade 3: 40 kg/m^2^ ≤ BMI.

Supplementary table 4 Full multivariable logistic model assessing the impact of PTS-Spo_2_ of 94-98% on hospital mortality

| Variables | Multivariable Logistic Regression Analysis | |
| --- | --- | --- |
|  | OR (95% CI) | P value |
| PTS-Spo_2_ of 94-98% (per 10% increase) | 0.968 [0.955, 0.981] | <0.001 |
| Age | 1.027 [1.024, 1.029] | <0.001 |
| BMI categories |  |  |
| Normal weight | 1 [Reference] |  |
| Underweight | 1.185 [0.999, 1.402] | 0.05 |
| Overweight | 0.886 [0.805, 0.975] | 0.013 |
| Obese grade 1 | 0.821 [0.735, 0.917] | <0.001 |
| Obese grade 2 | 0.832 [0.725, 0.954] | 0.009 |
| Obese grade 3 | 0.841 [0.737, 0.959] | 0.01 |
| Diagnosis |  |  |
| Cardiac arrest | 1 [Reference] |  |
| Respiratory | 0.296 [0.261, 0.335] | <0.001 |
| Sepsis | 0.363 [0.320, 0.411] | <0.001 |
| Cardiac surgery | 0.054 [0.042, 0.069] | <0.001 |
| Neurological | 0.427 [0.372, 0.490] | <0.001 |
| Cardiovascular | 0.271 [0.230, 0.318] | <0.001 |
| Trauma | 0.448 [0.370, 0.542] | <0.001 |
| Gastrointestinal | 0.288 [0.236, 0.350] | <0.001 |
| Others | 0.250 [0.217, 0.288] | <0.001 |
| Diabetes mellitus (yes vs no) | 0.846 [0.774, 0.924] | <0.001 |
| TWM-Fio_2_ (every 10% increase) | 1.169 [1.146, 1.192] | <0.001 |
| TWM-pH |  |  |
| Normal pH | 1 [Reference] |  |
| Acidosis | 1.372 [1.254, 1.501] | <0.001 |
| Alkalosis | 1.154 [1.019, 1.304] | 0.023 |
| pH missing | 0.951 [0.861, 1.050] | 0.325 |
| TWM-Paco_2_, mmHg |  |  |
| 35-45 | 1 [Reference] |  |
| < 35 | 1.530 [1.386, 1.690] | < 0.001 |
| > 45 | 0.801 [0.722, 0.888] | < 0.001 |
| Paco_2_ missing | 0.907 [0.717, 1.150] | 0.417 |
| SOFA score (not including respiratory part) | 1.201 [1.185, 1.217] | <0.001 |

PTS proportion of time spent, Spo_2_ peripheral oxygen saturation, BMI, body mass index, TWM time-weighted mean, Fio_2_ fraction of inspiration oxygen, Paco_2_ partial pressure of arterial carbon dioxide, SOFA sequential organ failure assessment.

BMI categories: Underweight: BMI < 18.5 kg/m^2^, Normal weight: 18.5 kg/m^2^ ≤ BMI < 25 kg/m^2^, Overweight: 25 kg/m^2^ ≤ BMI < 30 kg/m^2^, Obesity grade 1: 30 kg/m^2^ ≤ BMI < 35 kg/m^2^, Obesity grade 2: 35 kg/m^2^ ≤ BMI < 40 kg/m^2^, Obesity grade 3: 40 kg/m^2^ ≤ BMI.

Supplementary table 5 Full multivariable logistic model assessing the impact of PTS-Spo_2_ of 99-100% on hospital mortality

| Variables | Multivariable Logistic Regression Analysis | |
| --- | --- | --- |
|  | OR (95% CI) | P value |
| PTS-Spo_2_ of 99-100% (per 10% increase) | 0.997 [0.986, 1.009] | 0.636 |
| Age | 1.027 [1.024, 1.029] | <0.001 |
| BMI categories |  |  |
| Normal weight | 1 [Reference] |  |
| Underweight | 1.196 [1.008, 1.416] | 0.038 |
| Overweight | 0.871 [0.791, 0.958] | 0.005 |
| Obese grade 1 | 0.791 [0.708, 0.883] | <0.001 |
| Obese grade 2 | 0.797 [0.694, 0.913] | 0.001 |
| Obese grade 3 | 0.796 [0.697, 0.908] | 0.001 |
| Diagnosis |  |  |
| Cardiac arrest | 1 [Reference] |  |
| Respiratory | 0.282 [0.249, 0.320] | <0.001 |
| Sepsis | 0.349 [0.308, 0.397] | <0.001 |
| Cardiac surgery | 0.052 [0.041, 0.066] | <0.001 |
| Neurological | 0.427 [0.372, 0.490] | <0.001 |
| Cardiovascular | 0.261 [0.222, 0.306] | <0.001 |
| Trauma | 0.454 [0.375, 0.549] | <0.001 |
| Gastrointestinal | 0.284 [0.233, 0.345] | <0.001 |
| Others | 0.245 [0.212, 0.282] | <0.001 |
| Diabetes mellitus (yes vs no) | 0.858 [0.785, 0.938] | 0.001 |
| TWM-Fio_2_ (every 10% increase) | 1.169 [1.146, 1.192] | <0.001 |
| TWM-pH |  |  |
| Normal pH | 1 [Reference] |  |
| Acidosis | 1.348 [1.232, 1.475] | <0.001 |
| Alkalosis | 1.171 [1.035, 1.323] | 0.012 |
| pH missing | 0.940 [0.851, 1.038] | 0.224 |
| TWM-Paco_2_, mmHg |  |  |
| 35-45 | 1 [Reference] |  |
| < 35 | 1.574 [1.425, 1.737] | < 0.001 |
| > 45 | 0.786 [0.709, 0.872] | < 0.001 |
| Paco_2_ missing | 0.940 [0.743, 1.192] | 0.605 |
| SOFA score (not including respiratory part) | 1.205 [1.189, 1.221] | <0.001 |

PTS proportion of time spent, Spo_2_ peripheral oxygen saturation, BMI, body mass index, TWM time-weighted mean, Fio_2_ fraction of inspired oxygen, Paco_2_ partial pressure of arterial carbon dioxide, SOFA sequential organ failure assessment.

BMI categories: Underweight: BMI < 18.5 kg/m^2^, Normal weight: 18.5 kg/m^2^ ≤ BMI < 25 kg/m^2^, Overweight: 25 kg/m^2^ ≤ BMI < 30 kg/m^2^, Obesity grade 1: 30 kg/m^2^ ≤ BMI < 35 kg/m^2^, Obesity grade 2: 35 kg/m^2^ ≤ BMI < 40 kg/m^2^, Obesity grade 3: 40 kg/m^2^ ≤ BMI.

Supplementary figure 1 Missing rate for variables extracted from the database.


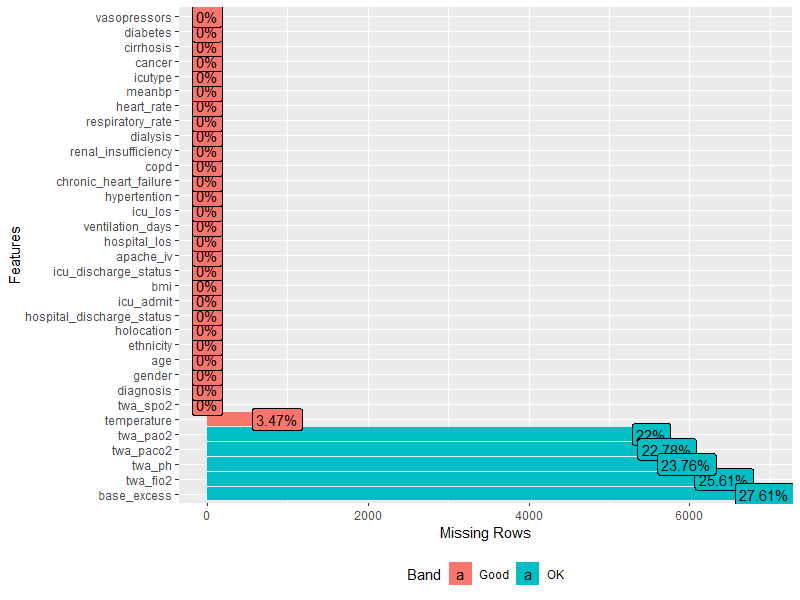


Supplementary figure 2 Distribution of proportion of time spent in different Spo_2_ categories


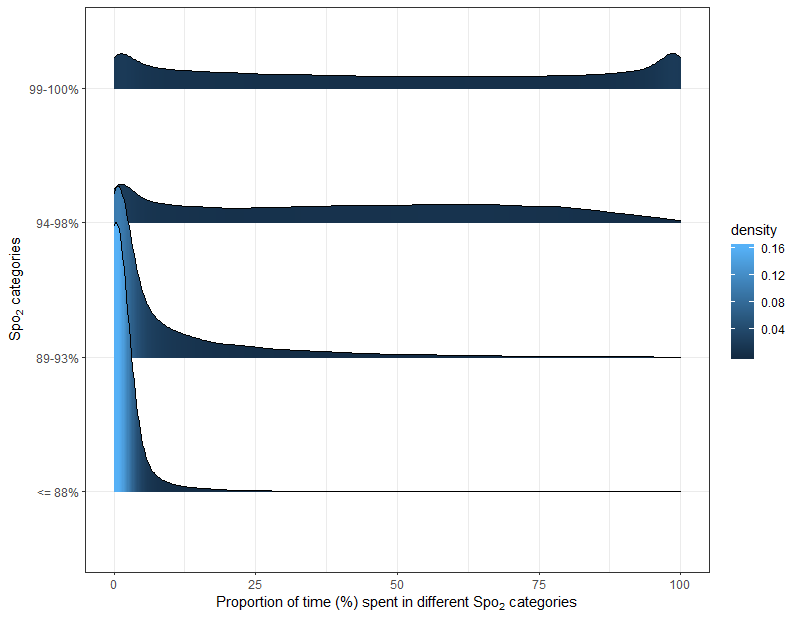


Supplementary figure 3 Interaction between PTS-Spo_2_ of <= 88% and 94-98% categories and BMI categories (obese and non-obese)


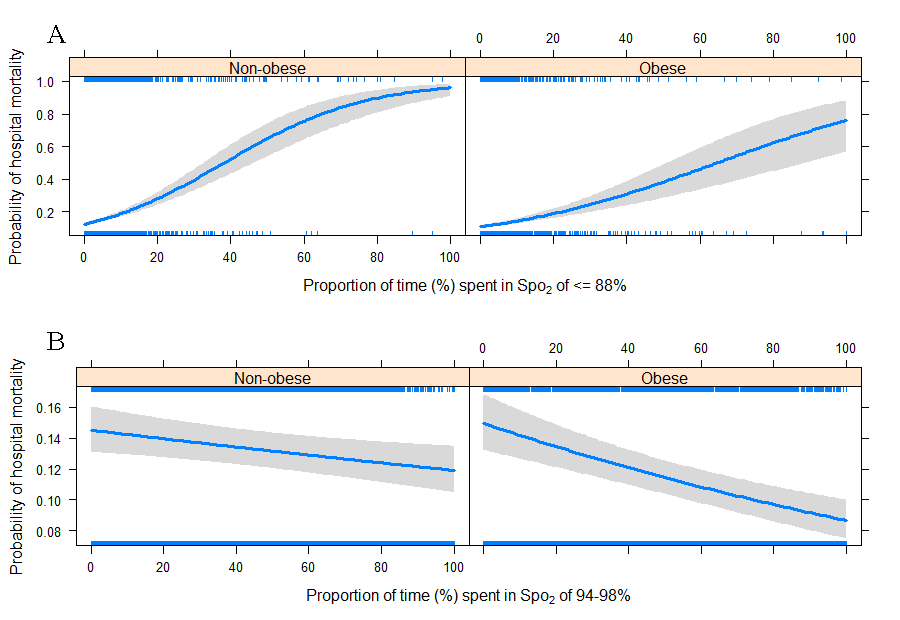


Upper (A): The PTS-Spo_2_ of <= 88% was associated with increased risk of hospital mortality outcome in both non-obese patients (OR 1.543; 95% CI 1.411-1.693; p < 0.001; per 10% increase) and obese patients (OR 1.361; 95% CI 1.240-1.495; p < 0.001; per 10% increase). Lower (B): The PTS-Spo_2_ of 94-98% was associated with decreased risk of hospital mortality outcome in both non-obese patients (OR 0.972; 95% CI 0.956-0.989; p = 0.001; per 10% increase) and obese patients (OR 0.948; 95% CI 0.928-0.969; p < 0.001; per 10% increase).

PTS-Spo_2_ proportion of time spent in peripheral oxygen saturation, BMI body mass index, OR odds ratio, CI confidential interval.

Supplementary figure 4 Scatterplot (using “jitter” method) of peripheral oxygen saturation (SpO_2_) recorded by monitor and nurse at the same time


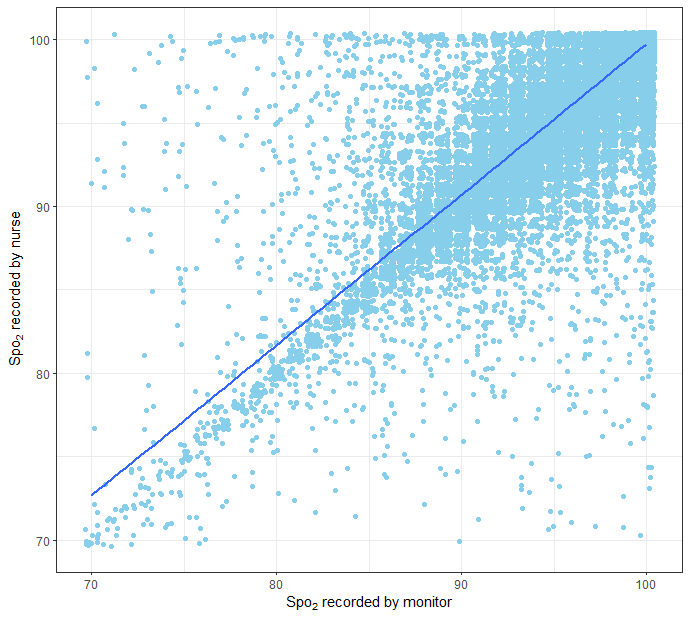


Blue line was linear regression curve. Correlation coefficient using “Pearson” method was 0.938.
